# Interpretive focus groups: a participatory method for interpreting and extending secondary analysis of qualitative data

**DOI:** 10.3402/gha.v7.25214

**Published:** 2014-08-18

**Authors:** Michelle Redman-MacLaren, Jane Mills, Rachael Tommbe

**Affiliations:** 1 College of Medicine and Dentistry, James Cook University, Cairns, Australia; 2 College of Healthcare Sciences, James Cook University, Cairns, Australia; 3 School of Health Science, Pacific Adventist University, Port Moresby, Papua New Guinea

**Keywords:** interpretive focus groups, secondary analysis, decolonizing methodologies, qualitative research, Papua New Guinea

## Abstract

**Background:**

Participatory approaches to qualitative research practice constantly change in response to evolving research environments. Researchers are increasingly encouraged to undertake secondary analysis of qualitative data, despite epistemological and ethical challenges. Interpretive focus groups can be described as a more participative method for groups to analyse qualitative data.

**Objective:**

To facilitate interpretive focus groups with women in Papua New Guinea to extend analysis of existing qualitative data and co-create new primary data. The purpose of this was to inform a transformational grounded theory and subsequent health promoting action.

**Design:**

A two-step approach was used in a grounded theory study about how women experience male circumcision in Papua New Guinea. Participants analysed portions or ‘chunks’ of existing qualitative data in story circles and built upon this analysis by using the visual research method of storyboarding.

**Results:**

New understandings of the data were evoked when women in interpretive focus groups analysed the data ‘chunks’. Interpretive focus groups encouraged women to share their personal experiences about male circumcision. The visual method of storyboarding enabled women to draw pictures to represent their experiences. This provided an additional focus for whole-of-group discussions about the research topic.

**Conclusions:**

Interpretive focus groups offer opportunity to enhance trustworthiness of findings when researchers undertake secondary analysis of qualitative data. The co-analysis of existing data and co-generation of new data between research participants and researchers informed an emergent transformational grounded theory and subsequent health promoting action.

Participatory approaches to research practice constantly expand and change (1, 2). Increasingly, researchers are being encouraged to undertake secondary analysis of qualitative data in order to maximise the utility of previous research studies. Secondary analysis of qualitative data is the use of existing data to develop new understandings of a research topic or methodology (3). By using existing qualitative data, researchers gain significant efficiencies in cost savings, time taken, and human resources required. In addition, secondary analysis of data addresses the ethical imperative to use data that may otherwise have lain dormant, and which in turn enhances outcomes from the impost placed upon research participants (4–6).

**Fig. 1:**
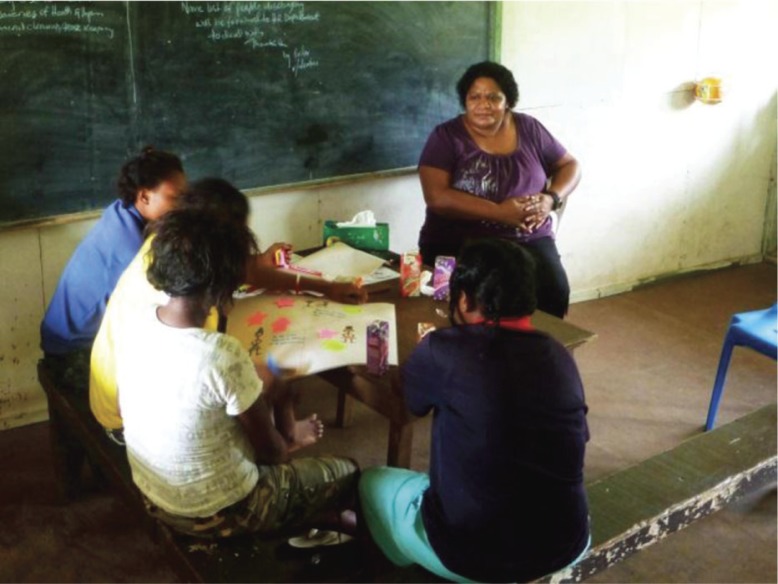
Young women draw their storyboards, supported by Rachael Tommbe (Reproduced with permission by M. Redman-MacLaren).

Despite the benefits of secondary analysis of qualitative data, there are a number of epistemological and ethical challenges for qualitative researchers. Rather than ‘collect’ data as quantitative researchers do, most qualitative researchers co-generate data in the context of a purposeful relationship (7, 8). This approach is underpinned by the researcher's epistemological and methodological position (8). It is possible the qualitative data were collected in a manner inconsistent with the researcher's epistemological and methodological position (9) and the quality of data may not be able to be verified (10). There are also ethical considerations when reusing qualitative data, including the challenge of providing ongoing anonymity for participants and the ability to obtain participant consent for the re-use of the data to address a different research question (11).

Secondary analysis of data can be conducted using different methods, depending upon the purpose of the research. Heaton (12) describes five types of secondary analysis of qualitative data. These are:

1. Supra analysis: examines new empirical, theoretical, or methodological questions.
2. Supplementary analysis: a more in-depth investigation of an emergent issue.
3. Re-analysis: data are re-analysed to verify or corroborate primary analysis of primary data.
4. Amplified analysis: combines data from two or more primary studies to compare or enlarge a sample.
5. Assorted analysis: combines secondary analysis of data with primary research.

There is a multiplicity of approaches and issues surrounding secondary analysis of existing qualitative data. In this article, we describe how we undertook secondary analysis of existing qualitative data about the acceptability of male circumcision by women in Papua New Guinea (PNG). We then describe how we facilitated primary research to explore how women understand, experience and manage male circumcision in PNG. Consistent with *assorted analysis* described by Heaton, we explain a two-step approach used to co-analyse existing qualitative data through story circles and then co-generate primary data through storyboarding. This approach resulted in data that informed an emergent transformational grounded theory and subsequent health promoting action. Below we expand previous descriptions of interpretive focus groups. We also describe modifications made to the research methods, consistent with participatory and decolonizing approaches to research practice.

## Interpretive focus groups

Focus groups are commonly used in qualitative research to explore and construct knowledge about a phenomenon with research participants in small groups (13–16). In health and development research, the researcher typically leads focus group discussions, with recordings of participant contributions made using audio recording devices and/or by having an observer in the focus group write down the contributions of participants (15). Specific focus group methods reflect the epistemological position of the researcher. Participatory and power-sharing approaches to focus group facilitation have been described by feminist researchers (17), action researchers (1, 18), decolonizing (19–21), and indigenous researchers (who also describe variations on focus groups such as conversational method, talking circles or research-sharing circle methods) (22, 23). One specific approach to focus group facilitation, originally described by feminist researchers, is the *interpretive focus group* (24, 25). Interpretive focus groups are facilitated with groups of people who have similar characteristics, brought together for their specific knowledge or experience to analyse data generated by others in a similar socio-economic setting (26, 27). Interpretive focus groups with participants of similar educational and/or cultural backgrounds reduce the risk of ‘missing the mark’ and increases trustworthiness of interpretation of the research findings (24). Below we describe an expanded two-step method of interpretive focus groups facilitated to: 1) analyse data from an existing data set using story circles and 2) co-generate new knowledge using storyboards. This paper expands interpretive focus group method by describing an iteration informed by participatory and decolonizing research methodologies. This expanded method informed the development of a transformational grounded theory and subsequent health promoting action to address human immunodeficiency virus (HIV) risk with women and men in PNG (28).

## The research context: Papua New Guinea

Papua New Guinea (PNG) is a diverse middle-income, Pacific Island nation of 6.8 million people who gained independence from Australia in 1975. In this hyper-diverse nation, over 800 languages are spoken and the rural majority (85%) live a predominantly subsistence lifestyle. PNG faces many health and development challenges, one of which is how to respond to HIV. In PNG, HIV is predominantly heterosexually transmitted and affects approximately 0.5% of the population, with some regions and populations (such as women) more affected (29). Evidence from three large randomised controlled trials in Africa showed a reduction in HIV acquisition of up to 60% for heterosexual men (30–32). Male circumcision is now being researched as a HIV prevention option in PNG, where male circumcision (the full removal of the foreskin from the penis) is only practiced by men in a small number of specific cultural groups (33). Given the low social and educational status of women in PNG (34), it is important to understand the implications of male circumcision for women. This will reduce the risk of negative, unintended consequences that may result from proposed health prevention programs, such as making it harder for women to negotiate safe sex. The doctoral research of MRM (Author 1) explored how women understand, experience, and manage the outcomes of male circumcision, building upon an existing research agenda investigating the acceptability and feasibility of male circumcision for HIV prevention in PNG (33, 35–37).

This grounded theory study drew upon an existing data set that included quantitative and qualitative data from a large mixed methods, multi-site male circumcision study, with 861 male and 519 female participants (33). Before undertaking doctoral research, MRM was project manager for this study (2010–2012) and RT an investigator on the study. RT had led many of the original interviews and focus group discussions and was familiar to some of the women who later participated in this subsequent study.

### Design: interpretive focus groups

Initially, MRM theoretically sampled data from individual interviews and focus group discussions from the existing data set. Rich interviews and focus group transcripts were imported and analysed using the computer software program, *NVivo* (Version10) with grounded theory data analysis methods employed, including initial, intermediate and advanced coding (8). Preliminary categories were developed from the existing data. Portions or ‘chunks’ of data that exemplified the developing categories selected were printed and laminated in multiple copies to be discussed by women in interpretive focus groups.

Seven interpretive focus groups were co-facilitated with 64 women at one urban university site and one rural site in PNG, both of which were sites in the previous multi-site male circumcision study (33). Theoretical sampling methods were employed to select participants, consistent with grounded theory methods (8). At the beginning of each interpretive focus group, researchers facilitated introductions, discussed the purpose of the focus group, and sought consent from the women to participate in the research. The groups were facilitated by MRM (an Australian researcher) and RT (a PNG researcher) in the languages of PNG Tok Pisin or English (for a detailed examination of cross-language research as enacted in this study, refer to Redman-MacLaren et al., forthcoming). Prior to discussing the data, a confidentiality agreement was made with women, based on an adapted agreement developed by Pittaway & Bartolomei:

‘Confidentiality means that we all promise not to discuss anything we hear in this group without the permission of the person who tells the story. It is a *promise* that we give each other, including facilitators and participants. If we agree to this, then we learn to *trust* each other and discuss things openly, because we all know it will not be spread around the community or used in reports without permission’ (emphasis used in interpretive focus groups) (38).

Human Research Ethics Committees of Pacific Adventist University (Papua New Guinea), James Cook University (Australia), and National AIDS Council Secretariat of PNG provided ethics clearance for this doctoral research.

## Results

### Step One: Interpreting data ‘chunks’ in story circles

Researchers invited women to discuss portions of data or data ‘chunks’ that had been identified during initial analysis of the data. The discussions took place in smaller story circles (typically 2–4 women) within the larger interpretive focus group. Four data ‘chunks’ were provided to women in English and/or Tok Pisin on two A4-sized pages. One page included two data ‘chunks’ about adult male circumcision and the other page included two data ‘chunks’ about infant male circumcision. Women discussed the data in story circles in a way they themselves determined. This process evoked a sharing of personal experiences – women discussed their interpretation of the data, their personal positions in relation to the data, and shared much laughter as well. The atmosphere in the groups was comfortable as women discussed their own stories, or accounts they could relate to, and this ignited further discussion. A spokeswoman from each story circle was then invited to share the ‘big ideas’ that had emerged from the story circle with the larger interpretive focus group. The story circles within the interpretive focus groups and this broader discussion were audio recorded using multiple digital audio recorders. In addition, notes from the whole-of-group discussion were handwritten by RT.

### Step Two: Storyboarding

Following the whole-of-group discussion, women were again invited to work in their story circles to extend their ideas about the data using storyboards. Storyboarding is a technique used in the visual arts that has recently been adapted for use in community development and participatory research (38). Women drew storyboards based on their discussions about the data ‘chunks’, focused by a set of questions developed by MRM during the initial coding of the existing data set. The questions were:

1. What is happening (how, who, where, when)?
2. What is the outcome for men?
3. What is outcome for women?
4. What needs to happen next?

Women drew pictures on their storyboards on large sheets of paper using crayons, felt pens, highlighter pens, and pencils (Figure 1). Various sized drawings emerged and women organised their information and drawings differently – some women drew only pictures and some drew a combination of pictures and words. Some women started drawing immediately, other story circles took more time to discuss the questions first and then draw their considered responses. Once again, a spokeswoman from each story circle shared the drawings and an explanation with the whole interpretive focus group, which often led to extended discussions. The whole-of-group discussions were audio recorded and later transcribed. Refreshments were provided at all groups.

Following the facilitation of interpretive focus groups at the two sites, MRM analysed the storyboards, the audio recordings, and handwritten notes. Use of grounded theory data analysis methods including constant comparison, memoing and initial, intermediate and advanced coding led to a tentative transformational grounded theory. The developing theory was then discussed in detail during a second round of discussions at the two field sites with women who had participated in the interpretive focus groups. In addition, the developing theory was discussed with relevant stakeholders such as health workers, company managers, and employees of non-government organisations. Final modifications were made to the grounded theory and first steps of the requested health promoting action were taken. This has included sexual health awareness sessions (39).

## Discussion

This two-step approach to interpretive focus groups was relevant for these people who live in a collective culture. Most women have low literacy levels, live in a postcolonial context, and many were involved in sensitive, sexual health research for the first time. Interpretive focus groups were a participatory way of undertaking a secondary analysis of existing data. This power-sharing approach is consistent with decolonizing methods when working across cultures and goes some way to addressing the dilemma of a researcher from a different cultural background interpreting existing data on ‘behalf’ of others. This two-step approach to interpretive focus group facilitation is a research method consistent with collective approaches to meaning-making that occur regularly in Pacific island and indigenous communities (19).

Focus groups are often designed in a top–down manner, with participants carefully sampled and numbers restricted to a recommended number (18). However, consistent with the participatory and decolonizing approaches, combined with the lived reality of research in the Pacific, the interpretive focus groups, we facilitated a more bottom-up approach. The sizes of groups varied according to the local social, cultural, and physical conditions. The number of women in the interpretive focus groups ranged from 4–15 women. On one occasion (30 July, 2013), MRM and RT arrived at a village in a rural area expecting to facilitate individual interviews, consistent with the research ‘design’. Instead, we were greeted by a group of women (n=8) ready for a collective discussion. We responded by facilitating an adapted focus group discussion (unfortunately, we were not prepared to facilitate a group and did not have our storyboarding materials with us). Being responsive and devolving power in groups requires researcher flexibility while being consistent with research principles and ethics. Researchers using this expanded interpretive focus group method can enable leadership and co-participation within story circles and the broader interpretive focus group, which can produce new understandings of existing data and generate new primary data.

Storyboarding in interpretive focus groups is a visual research method that encourages a different kind of participation and an additional way of communicating about sensitive sexual health issues. In this case, storyboarding stimulated succinct, targeted representations of women's knowledge and experience of male circumcision, beyond that possible by words alone (38). Storyboarding was therefore used to enable a visual representation of the data appropriate for cultural expressions in this Pacific context. Visual methods, including storyboarding, have also been used in other indigenous research (40, 41).

A participatory approach to interpretive focus groups reduces power differences between researcher and research participant (co-researcher), which is critical in a postcolonial context (1, 19). In PNG, white (in particular, white Australian) researchers have typically been seen as a continuation of the former colonial system. This approach to focus group facilitation centralises ‘story’ as a key medium for communicating meaning-making about the existing data, consistent with decolonizing methodologies (22). The groups’ agenda can be directed by participants on the micro level, increasing the likelihood of participation in the research and potentiating the goal of co-generating results that reflect the understandings of participants.

One major benefit of this two-step approach to interpretive focus groups was the way it enabled discussion about the sensitive sexual health subject of male circumcision and the implications for women in PNG. Secondary analysis of the existing data showed many women know a lot about male circumcision but few are culturally or socially sanctioned to speak about it. Discussion about sensitive data in small story circles resulted in a ‘gentling’ into the sensitive sexual health topic – women became more comfortable discussing the topic in safer, small story circles before it was discussed in the large group. For those not comfortable discussing the topic in a larger group, their opinions and experiences were still represented by their story circle spokeswoman and audio recorded for future analysis.

## Limitations of interpretive focus groups

There are a number of limitations to the methods described above. The two-step interpretive focus groups relied heavily on the researcher's analysis of existing qualitative data (in this case MRM). MRM chose the data included in the data ‘chunks’. Participatory action researchers typically analyse data generated with participants, not in isolation from them (1). One way of reducing the centrality of a specific researcher could be to undertake data analysis as a research team, with researchers from the country in which the data were collected. In this case, MRM and RT had previously examined and reported preliminary thematic findings from this data set (42) and thus data were not analysed in isolation of knowledge of social and cultural interpretations.

A further limitation of the method described is the small amount of data analysed by women in the interpretive focus groups when compared with the amount of qualitative data in the existing data set. The overwhelming majority of analysis of the existing qualitative data was conducted by MRM, with support from JM and RT, not the women participating in interpretive focus groups. However, the proportion of data being examined by the participants in interpretive focus groups should be weighed up against their other commitments and challenges (in this case, women who live a largely subsistence lifestyle and have limited literacy).

If the groups had more time, we would have negotiated our own confidentiality agreement with women rather than imposing one. Using an existing confidentiality agreement was a deliberate compromise given the constraints and context of the study. Many of the limitations experienced in this study reflect the competing demands and lived reality of participatory research in other contexts (18).

## Conclusions

This paper describes an approach that extends the interpretive focus group method previously described. Women discussed ‘chunks’ of existing data in story circles and then built upon this analysis, generating new (primary) data using storyboarding. The approach is consistent with participatory action research methods and power-sharing, decolonizing research methods, especially appropriate to Pacific islands and other indigenous research contexts. The extended interpretive focus groups enabled co-analysis of existing data and co-generation of primary data that informed an emergent transformational grounded theory and subsequent health promoting action.
